# HIV self-testing values and preferences among sex workers, fishermen, and mainland community members in Rakai, Uganda: A qualitative study

**DOI:** 10.1371/journal.pone.0183280

**Published:** 2017-08-16

**Authors:** Virginia M. Burke, Neema Nakyanjo, William Ddaaki, Caitlin Payne, Naadiya Hutchinson, Maria J. Wawer, Fred Nalugoda, Caitlin E. Kennedy

**Affiliations:** 1 Department of International Health, Johns Hopkins Bloomberg School of Public Health, Baltimore, Maryland, United States of America; 2 Rakai Health Sciences Program, Kalisizo, Uganda; University of Cyprus, CYPRUS

## Abstract

HIV self-testing may encourage greater uptake of testing, particularly among key populations and other high-risk groups, but local community perceptions will influence test uptake and use. We conducted 33 in-depth interviews and 6 focus group discussions with healthcare providers and community members in high-risk fishing communities (including sex workers and fishermen) and lower-risk mainland communities in rural Uganda to evaluate values and preferences around HIV self-testing. While most participants were unfamiliar with HIV self-testing, they cited a range of potential benefits, including privacy, convenience, and ability to test before sex. Concerns focused on the absence of a health professional, risks of careless kit disposal and limited linkage to care. Participants also discussed issues of kit distribution strategies and cost, among others. Ultimately, most participants concluded that benefits outweighed risks. Our findings suggest a potential role for HIV self-testing across populations in these settings, particularly among these key populations. Program implementers will need to consider how to balance HIV self-testing accessibility with necessary professional support.

## Introduction

Global efforts to control HIV have recently focused on high-prevalence geographic areas and populations perceived to be fueling HIV epidemics [1–5]. Fishing communities in sub-Saharan Africa are an example of such settings. They are frequently characterized by unique social and economic factors that contribute to high HIV prevalence, including high rates of transactional sex and sex work [6–8], mobility and migration [9, 10], a “culture of risk denial or risk confrontation” associated with an often dangerous industry [11], social marginalization [9], alcohol use [9, 12, 13], and limited access to health services [7, 9, 11, 12].

HIV self-testing (HIVST) is a promising approach to expanding HIV testing services and may have particular benefit in these unique environments [14], which are often underserved by traditional HIV testing services. HIVST is a process in which a person who wants to know his or her HIV status uses a kit to collect a specimen, performs a test (generally a rapid diagnostic test), and interprets the test results for himself or herself; positive results require confirmation testing at a health facility [14]. However, there has been little research on HIVST in high-prevalence geographic areas or with key populations in sub-Saharan Africa [15]. A recent systematic review of values and preferences related to HIVST conducted to inform World Health Organization guidelines identified 90 peer-reviewed articles worldwide [14]. Twenty-five were conducted in sub-Saharan Africa, but all were limited to general populations [14]. The majority of participants in these studies reported high acceptability of HIVST, but some voiced concerns about misuse [16–20].

Only three studies have explored perceptions of HIVST among sex workers—each from Puerto Rico, China, and Kenya [21–23]. In Puerto Rico, male and transgender female sex workers voiced concerns about confidentiality and triggering violence with clients [21]. In China, sex workers reported high acceptability of oral HIVST kits, but doubted the accuracy of the results [22]. In Kenya, secondary HIVST kit distribution to sexual partners of female sex workers was relatively successful, as 75% of participants distributed self-tests to their primary partners and 81% distributed more than one self-test to clients [23]. Provision of self-tests in this study also resulted in high levels of couples testing and significant changes in reported sexual risk behavior [23]. No studies have been conducted among fisherfolk, despite the high HIV burden among these communities. Furthermore, despite global efforts to focus HIV resources on high-risk populations, we do not know how values and preferences around HIVST may differ among high-risk groups compared to those of the general population.

Fishing communities along Lake Victoria in Rakai, Uganda have high HIV burdens, with a median HIV prevalence of 42% (range 38–43) [12], several times the Ugandan national average of 7.1% [24]. High HIV prevalence in these communities may be related to a combination of unique factors related to the fishing economy, including significant stigma, frequent unprotected sex, risk denial, and substance use [25–27]. Sex work is prevalent and individuals report higher rates of HIV risk behaviors compared to individuals in nearby mainland areas, including having more than one sexual partner in the past year and using alcohol before sex [12]. In this setting, we sought to qualitatively examine values and preferences related to HIVST among community members and health care providers in both mainland and high-risk fishing populations, including with sex workers and fishermen, in Rakai District, Uganda.

## Methods

Ethical approval for this study was received from the Western Institutional Review Board (20151088) and the Uganda Virus Research Institute Ethics Committee (GC-127-16-10-525). Prior to enrollment into the study, all recruited individuals provided written informed consent for participation. We conducted in-depth interviews (IDIs) with 12 health care providers (clinicians and HIV counselors) and 21 community members from both high-risk fishing communities and low-risk rural mainland communities in Rakai, Uganda (Table 1). Participants from fishing communities were purposefully selected to include sex workers and fishermen. Additionally, we conducted 6 focus group discussions (FGDs), with a total number of 55 participants, stratified by sex, location, and age (Table 1), to examine group discourse and social norms related to HIVST in both settings. Individuals were recruited through initial seeds provided by Rakai Health Sciences Program (RHSP) community health workers or through participants in the Rakai Community Cohort Study (RCCS) who had agreed to be re-contacted for future studies, with additional snowball sampling from initial participants. We followed an iterative process of data collection and analysis, ending recruitment when we felt data saturation was reached.

**Table 1:** IDI and FGD participants by location, group, and sex.

Study Participants		Fishing Communities	Mainland Communities	Total
**IDIs**	Community Members: Men	6 (fishermen/boat owners)	4	10
	Community Members: Women	8 (sex workers)	3	11
	Health Care Providers	3	9	12
	Total	17	16	33
**FGDs**	FGD Participants: Men	20 (2 groups)	10 (1 group)	30
	FGD Participants: Women	7 (1 group)	18 (2 groups)	25
	Total	27	28	55

Eligible participants were invited to participate in an interview or focus group at a time and location of their convenience. Before starting data collection, interviewers explained the purpose of the study and obtained written informed consent. Interviews and FGDs were conducted in Luganda or English and audio-recorded with permission from participants. Questions explored perceived positive and negative aspects of HIVST and implementation preferences. Participants were compensated with 10,000 Ugandan shillings for their time and transport costs.

IDIs and FGDs held in Luganda were translated by experienced graduate research assistants into English. All data was transcribed, coded and analyzed using a team-based matrix approach adapted from Framework Analysis [28]. Matrices allowed investigators to summarize and compare the data based on participant characteristics including sex, occupation, age, marital status, and location. Working collaboratively, several team members first read through the transcripts, then discussed and developed a set of codes to be used in the matrices that reflected both the original research questions and emerging themes. We then summarized each participant’s response for each code and included illustrative quotes. The matrices were completed by two team members independently for a set of sample transcripts, then discussed and refined. The finalized matrix was then used to code all other transcripts. In conjunction with the coding process, investigators wrote memos to develop conceptual categories and to track emerging insights on the data.

## Results

Table 1 presents the number of IDI and FGD participants by location, group, and sex. We conducted interviews with 10 men, 11 women, and 12 health care providers. Of these, approximately half were in fishing communities, where all men were fishermen or boat owners and all women were sex workers, and half in mainland communities, where occupation was not recorded. Among the participants who chose to self-report their HIV status, there was a mix of HIV-positive and HIV-negative individuals. More female sex workers reported known HIV-positive status than other participants. As there were no major differences between FGD and IDI participant responses, results from both data collection activities are combined in the themes presented below.

Most participants had never heard of HIVST. One mainland woman reported hearing of a school-based self-testing program in Tanzania, where students could test themselves privately and then do a repeat test with a counselor if warranted. Another female sex worker reported she had previously attempted to use an HIVST kit with her boyfriend who obtained it from the army, but due to what she described as mistakes in execution with the buffer, they were not able to obtain results. In this case, the sex worker knew she was HIV-positive and her boyfriend had asked her to test before having sex; when the test failed, she reported that they did not have sex, but she said she would have used it as a way to disclose her status had the test worked.

After being presented with the idea of HIVST, most participants felt it could have significant benefits, although others had serious concerns. These perspectives are outlined below.

### Perceived benefits

#### Privacy

Many participants (sex workers, fishermen, and health care providers) said HIVST would allow individuals to test in private and keep their results entirely confidential. A few sex workers liked the ability to keep positive results private until they could persuade their husbands to test, avoiding potential domestic violence. Many participants also referenced the ability to avoid stigma and community gossip associated with testing at health centers.

#### Avoid fear of health clinic

Both sex workers and health care providers in fishing communities frequently referenced patients’ fears of attending health clinics and suggested HIVST could enable patients to obtain preliminary results without visiting a clinic. This comment was less common in responses from mainland participants. Some discussed how HIVST might provide time to process and gather personal strength before seeking confirmatory testing at the clinic.

It helps me to know my status because I might fear to go to the clinic for HIV testing. I may not want to be tested by another person yet when I am alone I can take the initiative to bleed myself and conduct a self-test. When I find out that I am HIV positive, it can give me the courage to go to the hospital

*—Female Sex Worker*, *Fishing* Community

#### Convenience—Avoid transport time & money

Although positive HIVST results ultimately require a confirmatory clinic visit, the ability to obtain preliminary results without paying to travel to another town was a commonly cited perceived benefit. Participants said they were often away when mobile testing services visited their communities or they lacked time to travel to a facility. HIVST would enable testing on their own schedules.

*Self-testing saves money. Some of us are far away from the health centers. The money that you would have used to go to the health center, you use it to buy your loved one something to eat*.

–*Male Community Member, Mainland Community*

#### Testing before sex

Participants from both fishing and mainland communities suggested HIVST kits could be used to test new partners before engaging in sexual relationships. Many sex workers referenced their inability to consistently insist on condom use with clients due to financial incentives, threats of violence, or pierced condoms. A few sex workers suggested HIVST might enable them to test clients before having unprotected sex, as this participant described:

*… a man may come and tell you that he does not have HIV pretending that he tested HIV negative. Mentioning this, he has neither shown you his result nor came with them at your place. He does this purposely to instill in you the idea of having sex without a condom. So if you have that kit, you can test him since he said that he does not have HIV and confirm what he said*.

–*Female Sex Worker, Fishing Community*

#### Earlier linkage to care

Mainland health care providers suggested HIVST could bring more HIV-positive individuals into the clinic to initiate ART at earlier stages of disease, while treatments are most effective. They explained that currently, many patients do not seek clinic-based testing and care until their disease has progressed to an advanced stage.

### Perceived barriers

#### Absence of professional support

The most commonly voiced perceived barrier to HIVST was the absence of a health professional during the testing process. Participants from all groups strongly questioned the risks associated with not having health provider assistance and/or counseling before and after testing. Many fishing community participants worried they would misinterpret the instructions. A few mainland participants were concerned about receiving a false positive result.

One mainland health worker worried that people would misinterpret negative HIVST results to mean they would always be negative. Without counseling, he worried that individuals may not realize they could be testing within an incubation “window period”, nor would they receive counseling against high-risk behaviors that might result in seroconversion if continued.

Many participants from both fishing and mainland communities also feared people might commit suicide if they received positive HIVST results without formal support from a health care provider or counselor. As one fisherman said,

Especially for a person who has never tested for HIV … there is always a lot of tension and stress that goes on in his or her mind. Now, imagine if the test result is positive and this person is alone, whatever comes in his mind is what he/she does. These days we hear people killing themselves. Now if one finds out that he/she is HIV positive, do you see how he/she can commit suicide?

—*Male Community Member, Mainland Community*

#### Improper disposal of HIVST kits

Some participants from both mainland and fishing communities voiced concerns about improper disposal of HIVST kits after use, particularly if testing involved a blood draw. A few fishing community members feared others might discover the kits, leading to gossip or domestic abuse. Several mainland community members and health care providers worried that used kits might accidentally infect others, especially children, if inappropriately discarded. One health care worker said,

*With regard to infection control, there is a way we dispose our sharp materials which are being used in the health setting. This helps us not to prick another person after being used. I have a fear that, for example, if that testing kit involves pricking and use of blood, a person can prick himself and puts the injection aside waiting for the results. When he gets results which are HIV positive, someone can get a mental confusion and forgets that he left the picker aside. Therefore, a needle can prick other members around him … This may lead to transmission of HIV and other infections*.

—*Health Worker, Mainland Community*

#### Poor or delayed linkage to care

Some participants from mainland and fishing communities expressed concerns that individuals who test positive with an HIVST kit would not seek confirmatory testing at a health facility. A couple of mainland community members predicted that people would be too emotionally overwhelmed by positive results to seek confirmation at the clinic. Others referenced alternative illness etiologies, suggesting some individuals would attribute their positive HIVST result to non-medical causes and thus not seek care.

A few sex workers said that HIVST would only delay access to ART compared to testing at a facility, after which one could immediately access treatment. One woman saw no value in HIVST, since one still needed to visit the clinic to receive a diagnosis and access treatment.

#### False claims of HIV-negative status

Multiple mainland community members expressed concern that HIVST would lead people to keep their HIV-positive status a secret and/or lie to their partners about their HIV status. They implied that a diagnosis at a health facility is more public and irrefutable, but a diagnosis by HIVST kit could be denied, as suggested by this focus group interaction:

*Participant 1: But I might test myself in hospital and I go back and ask my husband to test himself, but he tells me to bring him that kit because he doesn’t have time to go to hospital. His results may turn out positive but he will lie to me and say that he is okay when he has thrown away the used thing with evidence*.

*Participant 2: That is very true. If he found he was sick, he may never tell you, but if you go to the health worker they will give him a slip showing his results*.

–*Female Community Members, Fishing Community FGD*

#### HIVST kits used to harm

Though a unique response, one sex worker feared test kits could be used as weapons—she felt many clients, particularly fishermen, were “perverted” and had “hard, murderous hearts”, and she worried that sharp elements of a self-test kit could be used to harm sex workers.

### Proposed solutions to barriers

While a small number of participants could not identify any solutions to overcome the perceived barriers (particularly violence), the majority felt education and counseling before HIVST kit purchase would improve accuracy and support mental health of testing individuals. In fact, nearly every participant articulated something like this fisherman:

*I would like to be taught how to use the kit. I would like the health workers to explain to me the problems I may face when using the kits. They should also explain to me the problems I may face if I don't use the kit*.

–*Fisherman, Fishing Community*

In this counseling, individuals would learn how to properly administer and interpret the self-test, and discuss what to do if they test positive. Health care providers discussed the need to explain returning to the clinic for confirmation tests; one suggested individuals provide contact information for follow-up. Multiple participants suggested there be a phone number to call for assistance, if needed; another suggested instructions be provided in Luganda, the local language. Community members suggested that counseling might also mitigate the threat of self-harm after testing positive, and mentally prepare individuals to access treatment.

### Willingness to use and recommend HIVST to others

Almost all participants reported willingness to use an HIVST kit if provided and if educated sufficiently. The majority of sex workers said they would recommend HIVST to others, but mentioned their concerns described above. Two women fiercely disagreed, perceiving HIVST to be too risky. All fishermen and mainland community members said they would recommend HIVST to others. Of the health care providers interviewed, most would recommend HIVST to their patients, but emphasized the need for significant education beforehand. A couple of health care providers believed the risks outweighed the benefits and maintained that facility-based testing was necessary.

### Potential beneficiaries of HIVST

While many participants said they would recommend HIVST to anyone who needed to know their HIV status, they saw particular benefits for married couples, youth, and groups perceived as “high risk”, including sex workers, truckers, bar workers, fishermen, and motorcycle taxi drivers known as “boda boda men”. For married couples, participants said the privacy of HIVST could help avoid suspicions of infidelity when testing. School-aged youth and younger men were seen as benefitting because they were particularly sexually active and may not know their HIV status. Sex workers, bar workers, truckers, bus drivers, “boda boda men”, and fishermen were all identified by participants across communities as potentially benefitting from HIVST due to high-risk lifestyles often involving alcohol and multiple sexual partners. Sex workers thought HIVST could be very useful for other women in their profession, due to increased HIV risk and clients’ desire for unprotected sex.

*So when I pull out a condom, the customer asks me why I am getting a condom and asks me, "have I come to do sex with a condom or with you?" I then tell him that you don't know my HIV status and I don't know yours. He then says, "I don't have sex with a condom." … Most of my customers want to have sex with me without a condom. Others pierce the condom tips using their grown nails. Those are the problems we get. So I really recommend that sex workers use the HIV self-testing kits*.

—*Sex Worker, Fishing Community*

### Cost

Most participants said they would be willing to pay for an HIVST kit. When asked how much, responses ranged from 1,000 USH to 100,000 USH (US $0.29 to $29). The handful of participants who responded with the highest figures said they highly valued HIV testing and their personal health. However, most fishing community participants recommended between 3,000–5,000 USH (US $0.88-$1.47) and most mainland community participants recommended a maximum of 2,000 USH (US $0.59). Multiple health care providers believed patients would not realistically access kits unless they were free of charge. Several participants warned that high prices would incentivize forgery of HIVST kits.

### Distribution sites

Most participants preferred obtaining HIVST kits at health centers, ideally in their communities, and had mixed opinions about obtaining HIVST kits from other locations. At health centers, purchasers would be able to receive counseling from a health provider before testing. Additionally, many participants, particularly from mainland communities, stated that non-health facility locations (i.e. retail shops, pharmacies) were not qualified or equipped to distribute HIVST kits due to an inability to provide adequate counseling or storage, and concerns that shop owners may try to profit from kit sales.

*Many people are confident that there is privacy at the facility and they can freely talk to the health providers. … At the health facility these kits will be kept well and there is going to be good accountability. Furthermore, we can easily tell at the facility that the kits are either expired or about to expire and replace them. This is going to be better than when they are distributed through the shops or pharmacies who are after earning money*.

–*Health Worker, Mainland Community*

Participants who were willing to obtain HIVST kits at non-health facility locations valued the accessibility of these alternate distribution locations. Young males from both fishing and mainland communities expressed interest in having HIVST kits in lodges and bars like condoms are distributed, so that they are easily accessible outside of regular clinic hours or on short notice.

*If those kits are put in places like bars, hotels where different people go, I can get them. I can pretend as if I have come take a beer and then get a kit. I may have gone to the hotel to eat food and get it. You will not ask me what has brought me to the hotel*.

—*Fisherman, Fishing Community*

### Social dynamics

#### Stigma

Participants from both fishing and mainland communities discussed how social stigma associated with an HIV-positive diagnosis could influence HIVST use in both positive and negative ways. Some anticipated self-testing kits would face the same negative stigmatization associated with other HIV services. “The people may fear being seen in possession of these kits if they perceive others to stigmatize them”, said one mainland health worker. Others saw self-testing as a way to avoid or delay experiencing public stigmatization at health facilities. Multiple sex workers referenced the stigma associated with seeking care at HIV clinics and fears of being shamed by health workers.

#### Partnership dynamics

Discussions around HIVST and partnerships focused on disclosure and fears of abuse and abandonment. Female participants from fishing communities more frequently cited threats of domestic violence than did women in mainland communities, and many health care providers echoed this concern. Some specifically described the threat of violence if men blame their wives for infecting them, though this was not unique to self-testing.

*It will happen when people test themselves, find themselves with the virus and then blame their partners for spreading the virus to them. Then there are people who test themselves in this community, find out that they are HIV positive and then get scared of telling their husbands about their results because they will blame the for infecting them, and yet they may not necessarily be the ones who may have infected them. Yes, most men do not want to hear that a woman tested positive*.

—*Female Community Member, Mainland Community*

Partners could positively influence testing too, encouraging one another to test and supporting preventative behaviors. Finally, a few participants from both fishing and mainland communities hypothesized that as more people access and discuss self-testing services, others will increasingly accept self-testing as a feasible option.

## Discussion

This qualitative study describes perceptions of HIVST among both lower-risk mainland and high-risk fishing community members in Uganda (including sex workers and fishermen), populations that may benefit from self-testing programs following 2016 World Health Organization (WHO) guidelines recommending HIVST [14]. Participants discussed a range of benefits including privacy and convenience, noted potential barriers related to education and support, and suggested cost and distribution strategies if implemented in their communities. Many of the perceived benefits and barriers described by participants in this study align with findings from other studies on values and preferences related to HIVST globally [14] and among key populations (26). Recent systematic reviews found that barriers to HIVST uptake among key populations include doubts about accuracy, lack of provider assistance, and concerns about use, while facilitators include convenience and privacy [14, 15]. While the overall majority of participants concluded that the benefits of HIVST outweighed the risks, participant responses suggest HIVST may particularly address some of the economic and social barriers to facility-based HIV testing unique to fishing community settings.

Participants’ primary concern about HIVST was the lack of professional support if testing outside of a health care facility. A recent systematic review identified similar concerns about the risks of violence and nondisclosure from studies conducted among general populations in Africa [14], though reports from Nigeria [18], Kenya [19], and Zambia [17] suggest many participants anticipate the benefits of HIVST will outweigh these risks. WHO guidelines state that there is no evidence of any serious adverse events or harm as a result of HIVST, but that inclusion of risk mitigation and reporting strategies is still important [14]. There are several ways education and professional support could be incorporated into a self-testing model to alleviate some of these concerns. As some participants in this study noted, the most conservative option would be to distribute HIVST kits at health facilities where individuals could receive traditional pre-test counseling. However, this strategy may face the same challenges as existing facility-based services. Evidence from pilots in South Africa [29] and Malawi [30] support the feasibility of providing HIVST education outside of facility-based settings. If HIVST kits are available outside of clinics (e.g. at pharmacies, lodges, bars), self-testing information could be communicated by community health workers [20] or incorporated into existing health education campaigns, a strategy supported by the WHO [14]. Effective pre-test counseling in any form could support correct self-testing practices and accurate interpretation of results, emotionally prepare patients to receive results, and emphasize linkage to confirmation testing and treatment. However, this does not eliminate the need to create a more approachable healthcare environment for patients and decrease HIV stigma in these communities.

While most participants wanted to access HIVST kits at health facilities, young men and fishermen favored more convenient points of access such as bars and lodges. HIVST pilots in Kenya [31] and Zimbabwe [32] tested non-facility-based HIVST kit distribution strategies (distribution by sexual partners and community health workers, respectively) and both reported increased rates of kit use and successful testing. Studies from Kenya, South Africa, and the United States suggest the accessibility and privacy of HIVST may be uniquely effective at reaching previously unengaged populations, particularly men and youth [31, 33–35]. Since these groups have high rates of HIV and sexual risk behaviors [12], it may be advantageous to consider their specific preferences to expand coverage of HIV testing beyond existing systems.

The use of HIVST to learn a new partner’s HIV status before engaging in sex appealed to both men and women in this study. Female sex workers, in particular, saw this as a benefit since so many clients refuse condoms. The use of HIVST as a harm reduction strategy among populations who report inconsistent condom use has been explored in several U.S.-based studies [36–38]. Data from Kenya [31] and the United States [36, 39] indicate HIVST may be used as a screening tool for potential partners, and suggest individuals who receive a positive self-test result may be less likely to engage in condomless sex [31, 39]. However, sex workers whose clients receive negative HIVST results may be less likely to use protection with these partners. The 2016 WHO HIVST guidelines conclude the benefits of HIVST outweigh these potential risks, but advise that HIVST programs include clear messaging about potential risks of using HIVST in this way [14].

Participants in this study were not specifically asked about their preferences regarding oral swab testing versus whole-blood finger-prick testing, but several participants expressed concerns about sharp testing materials that could be used as weapons or accidentally infect others when discarded inappropriately. One can assume these risks would not be anticipated with oral swab testing. Other studies have generally suggested that people prefer oral swab to blood-based self-testing [15, 40], usually because it is pain-free [22, 41] and no blood is involved [22]. However, previous studies, including with sex workers, reported that some pilot participants question the reliability of these results (23). While oral specimen-based rapid tests have shown high positive predictive value in high-prevalence settings, they have slightly lower sensitivity and positive predictive value in low-prevalence settings [42]. Previous challenges interpreting rapid diagnostic tests in Rakai [43] suggest additional research may be needed to identify the most effective method of informing testers how to accurately complete and interpret HIVST results. Program implementers will need to consider these trade-offs when developing HIVST programs.

This study had several limitations. Although a few sources report informal availability of HIVST kits in some private clinics in Uganda [44], the majority of our participants reported never having heard of HIVST prior to this study. Interviews thus focused on hypothetical circumstances rather than direct experiences with HIVST, although interviewers did try to probe participants to describe the factors influencing their choices and explain any previous experiences relevant to their responses. Some meaning could have been lost in translating interviews from Luganda to English, although the research team that collected the original data was also involved with translation, interpretation and analysis. Despite these limitations, we believe we reached saturation of themes for our primary research questions and believe the findings of this study may be transferable to similar contexts.

With the new 2016 WHO recommendation for HIVST [14], we anticipate a greater expansion of self-testing programs globally. In Uganda, several HIVST pilot initiatives are underway, including two testing peer-distributed HIVST strategies among key populations–female sex workers in Kampala [45] and fisherfolk in Buliisa [46]—and another testing a partner-distributed strategy among women attending antenatal care facilities in the Central region [47]. The Ugandan Ministry of Health is currently considering introducing oral HIV self-testing kits, based on evidence from these pilot programs, to increase low uptake of HIV testing among men in particular [48]. Evidence from these pilots will continue to inform HIV self-testing scale-up in Uganda and around the world.

## Conclusion

Fishing community members often have high HIV prevalence and incidence, yet face financial and social barriers to facility-based services. The privacy and convenience afforded by HIVST may particularly benefit these key populations, although these same features may be problematic when associated with a lack of supportive counseling and linkage to care after an HIV-positive test result. Overall, nearly all participants in this study concluded that the personal benefits of HIVST outweighed the risks, that they would be willing to self-test, and that they would recommend self-testing to others. HIVST may be a unique tool to reach these groups, though program implementers will need to consider how to balance accessibility with necessary professional support.
